# Mosquito-borne viruses causing human disease in Fennoscandia—Past, current, and future perspectives

**DOI:** 10.3389/fmed.2023.1152070

**Published:** 2023-03-27

**Authors:** Lukas Wilkman, Clas Ahlm, Magnus Evander, Olivia Wesula Lwande

**Affiliations:** ^1^Department of Clinical Microbiology, Umeå University, Umeå, Sweden; ^2^Umeå Centre for Microbial Research, Umeå, Västerbotten, Sweden

**Keywords:** arbovirus, mosquito-borne virus, mobovirus, epidemiology, Fennoscandia, Sindbis virus

## Abstract

Five different mosquito-borne viruses (moboviruses) significant to human disease are known to be endemic to Fennoscandia (Sindbis virus, Inkoo virus, Tahyna virus, Chatanga virus, and Batai virus). However, the incidence of mosquito-borne virus infections in Fennoscandia is unknown, largely due to underdiagnosing and lack of surveillance efforts. The Fennoscandian moboviruses are difficult to prevent due to their method of transmission, and often difficult to diagnose due to a lack of clear case definition criteria. Thus, many cases are likely to be mis-diagnosed, or even not diagnosed at all. Significant long-term effects, often in the form of malaise, rashes, and arthralgia have been found for some of these infections. Research into mobovirus disease is ongoing, though mainly focused on a few pathogens, with many others neglected. With moboviruses found as far north as the 69^th^ parallel, studying mosquito-borne disease occurring in the tropics is only a small part of the whole picture. This review is written with the objective of summarizing current medically relevant knowledge of moboviruses occurring in Fennoscandia, while highlighting what is yet unknown and possibly overlooked.

## Introduction

During the last decade, emerging viral diseases have increased considerably in incidence in regions all over the world, many of these previously neglected as sporadically occurring viruses of limited importance to public health. Of these viral diseases, arboviruses (**ar**thropod-**bo**rne) have seen the greatest global spread in recent times, with dengue, chikungunya, Zika and yellow fever seeing unprecedented spread from 2015 onwards (1). The global burden of vector-borne viruses cannot be overstated; with 80% of the population at risk for one or more vector-borne disease, and already causing 17% of the global burden of communicable disease, including 700,000 deaths annually, their impact on human society is clear (1).

Arboviruses are defined as viruses replicating within arthropods and are then spread to vertebrate hosts. These viruses, belonging to a group of between 500 and 600 viruses including the families of *Peribunyaviridae, Phenuiviridae, Flaviviridae, Reoviridae, Rhabdoviridae*, and *Togaviridae*. Viruses belonging to *Peribunya-, Phenui-, Flavi-,* and *Togaviridae* are known to be transmitted by hematophagous (i.e., blood-feeding) insects and other arthropods ranging from ticks to mosquitoes (2–4). For example, most flaviviruses in Fennoscandia are transmitted by ticks with a key example being the tick-borne encephalitis virus (TBEV). This review focuses on the viruses spread by mosquitoes, known as **mo**squito-**bo**rne **viruses**, or moboviruses.

While the number of mosquito-borne diseases and their incidence in Europe is far lower than those of tropical regions, they are nevertheless a substantial contributor to infectious disease morbidity (5). With changing climates, future outbreaks may occur in areas where populations are immunologically naïve, and public health systems are unprepared (1, 3, 6, 7). Historically, mosquito-borne pathogens have been a major source of disease in Europe, with malaria being widespread as far north as Fennoscandia until the mid to late nineteenth century (8, 9). In more recent times, moboviruses belonging to the California encephalitis group of genus *Orthobunyavirus*, family *Peribunyaviridae* have been isolated as far north as the Finnmark region of Norway, in the village of Masi (69°26′N) (10). With appropriate, competent vectors found throughout Fennoscandia, it may only be a matter of time before mosquito-borne disease returns to be the major public health concern it once was in Fennoscandia, and currently is in more temperate regions.

Fennoscandia is the geographical peninsula that includes Scandinavia, in addition to Finland, Karelia and the Kola peninsula. This is distinct from Fenno-Scandinavia, which is simply Finland and Scandinavia. For the purposes of this review the term Fennoscandia, as well as the region it represents was used because of the relative ecological unity in the subarctic climate of the area in respect to which vectors are present, and which viruses they carry. This ecological unity would not be as clear if Iceland or Denmark were included, or Finland omitted.

The mosquitoes (*Culicidae*) have ever since they were first identified as the vectors of yellow fever (11), been at the center of medico-entomological research due to their significance as efficient vectors of both human and animal disease (4). Mosquitoes are very adaptable vectors, capable of thriving in a large variety of environments; from lakes to water-filled footprints, there’s hardly any aquatic habitat unsuitable for mosquito larvae (4). Large areas in Fennoscandia are covered by forests and there are a multitude of wetlands and water bodies. This in combination with a relative high precipitation makes the area favorable for mosquitoes (12). Finland features 43 different recorded blood-sucking mosquito species, and Norway 38. In Sweden there are around 50 different species, with most of the additional species being found further south than the southernmost regions of Finland and Norway (12–15).

In our changing climate, it is possible for invasive species of mosquitoes to spread disease previously unknown to that area, as was observed in Europe when the chikungunya outbreak occurred in Italy in 2007 (16). Further autochthonous cases have been detected in southern Europe since then, especially in areas where the invasive species *Ae. albopictus* has been established (7, 17).

As previously highlighted, there are many gaps in the present knowledge of the transmission of moboviruses, and the diseases they cause in humans. By furthering the research into these viruses, and the way they are spread, better diagnostic criteria and new treatment options may be developed. Through this, cases of human infection may be detected earlier, and morbidity as well as mortality reduced. Through improved early surveillance, case series may be prevented before they develop into outbreaks. Together, all these steps may lead us onto our ultimate goal, reducing patient suffering, by coming up with useful therapeutics and vaccines for the management and prevention of disease spread.

## Viral genetics and structure

The Fennoscandian arboviruses known to be human pathogens fall under the families *Peribunya*-, *Flavi*-, and *Togaviridae*. Of these, the ones spread by mosquitoes all fall under either the genera *Orthobunyavirus*, in the family *Peribunyaviridae*; or *Alphavirus*, the sole genus in the family *Togaviridae*, while the flaviviruses are transmitted by ticks. (18, 19). Accordingly, only these two genera will be further described in this review.

Alphaviruses are enveloped viruses with a single-stranded positive sense RNA genome of 10–12 kb housed in spherical virions, around 70 nm wide. They are characterized by their impressive host range, including vertebrate hosts such as humans, non-human primates, equids, birds, amphibians, rodents, and pigs, as well as sea mammals and fish. Sindbis virus is the sole member of *Alphavirus* found in Fennoscandia (2, 20).

**Figure 1:**
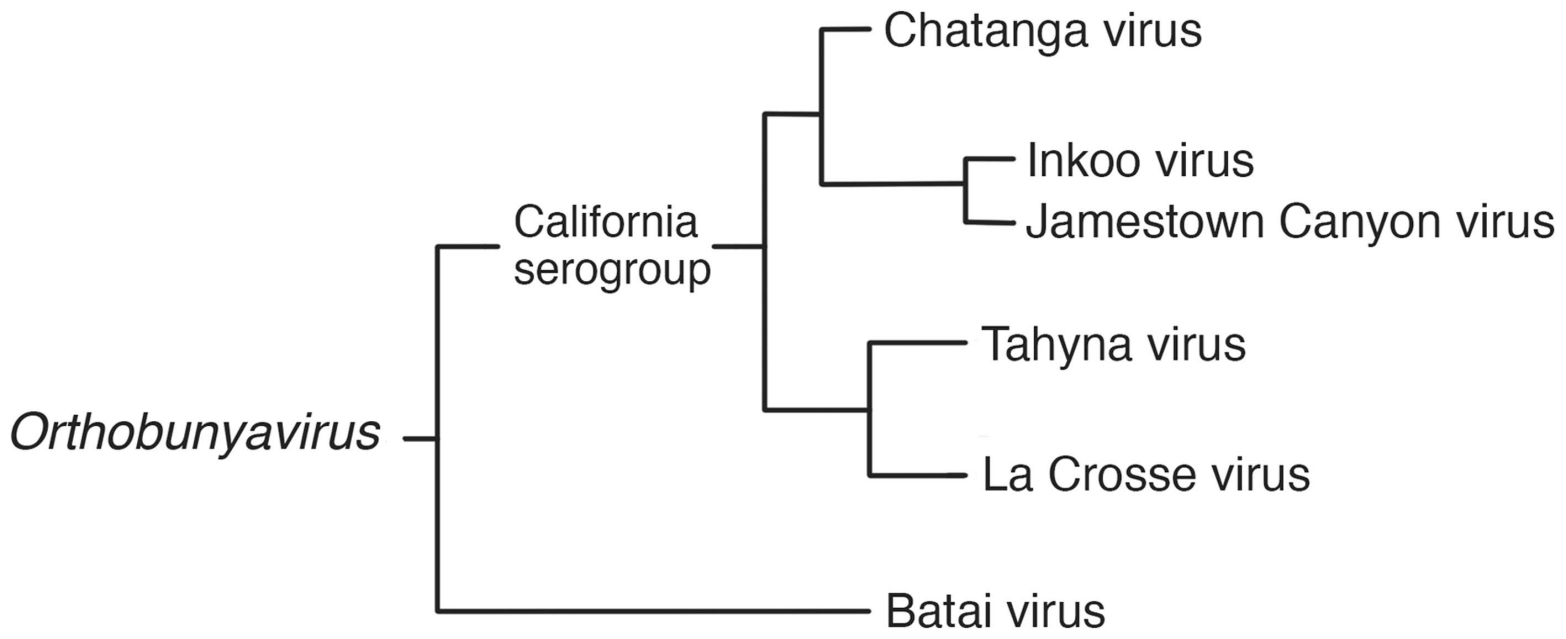
Phylogram of selected viruses from the *Orthobunyavirus* genus, including the three Fennoscandian moboviruses in the genus (19, 21, 23, 24).

Orthobunyaviruses have a segmented genome, with three segments of negative-sense RNA (S, M and L) of 12.4 kb in total; housed in enveloped, spherical virions of 80–120 nm in diameter. It is the largest and most diverse genus in the family, with a wide range of vertebrate hosts including squirrels, bats, rabbits, ungulates, sloths, and birds. The orthobunyaviruses found in Fennoscandia are Chatanga virus, Inkoo virus, Tahyna virus, and Batai virus. Of these four, all but Batai virus falls under the California serogroup, grouped together by their similarity to the prototypical California encephalitis virus (CEV) (21, 22). The phylogeny of relevant viruses in the genus *Orthobunyavirus* is summarized in Figure 1.

Regarding viral characteristics of transmission, the two genera differ in their method of genetic recombination. The positive sense single-stranded genomes of alphaviruses undergo frequent recombination, associated with genetic diversity (25). The segmented genome of *orthobunyaviruses* allow for reassortment to occur, while recombination is rare. This has been implied to play an important role in *Orthobunyavirus* emergence and virulence, especially though interspecies transmission. (26).

## Epidemiology and disease

### Sindbis virus

Sindbis virus (SINV) is the most clinically important of the Fennoscandic arboviruses (19), and is the sole member of the genus *Alphavirus* present in Fennoscandia. SINV was first isolated in 1952 near the village of Sindbis in Egypt, with the first case of human disease reported in 1961 (27). In Sweden, SINV was first isolated in the Swedish village of Ockelbo in 1982 and therefore called Ockelbo virus. It is very closely related to SINV-I, the only genotype out of six recognized (I-VI) associated with human disease (28), in both structure, pathogenicity, and antigenicity. It differs from the other SINV strains found in mainland Europe in that it is most closely related to the South African, suggesting that SINV was likely introduced to Fennoscandia by migratory birds, which have been observed to act as amplifying hosts to SINV, rather than spreading *via* mainland Europe (29, 30). The South African origin has however been challenged, due to a suggested origin in Central Africa found by Ling et al. (28).

The human disease caused by SINV, known as ‘Ockelbo fever’ (Sweden), ‘Pogosta disease’ (Finland), ‘Karelian fever’ (Russia), and generally as ‘Sindbis fever’. Other terms include “August–September disease” (*augusti-september-sjukan*) and “berry-pickers disease” (*bärplockarsjukan*, *bærplukkersyken*), named after the time-period and population in which it is mostly diagnosed (31). SINV causes occasional epidemics in Fennoscandia, characterized by a febrile maculopapular rash with myalgia and polyarthritis, of which the last symptom often persists the longest (32). Despite the avian SINV seroprevalence being widespread in Australasia and Africa, human SINV infection only appears clinically apparent in Northern Europe (mainly 60°–64°N) and South Africa, where the SINV-I genotype is dominant (27, 30, 33, 34).

SINV has an incubation period of 5–7 days before the onset of symptoms, after which IgM and IgG are detectable within 8 and 11 days respectively, resulting in a delay of 2–3 weeks between initial virus acquisition and the time a serological diagnosis can be made (35), at which point the extra articular symptoms have usually diminished. The articular symptoms may however persist for several months, in some cases even several years (36, 37). In Fennoscandia, SINV is mainly vectored by the ornithophilic mosquitoes *Aedes communis, Ae. cinereus, Ae. excrucians, Coquillettidia richiardii, Culex pipiens, Cx. torrentium*, and *Culiseta morsitans*. Additionally, it has also been found in *Anopheles maculipennis sensu* lato (18, 38–41). Of these species, *Ae. communis*, *Ae. cinereus*, *Ae. exrucians*, *Cq. richiardii* also readily feed on humans (42).

**Figure 2:**
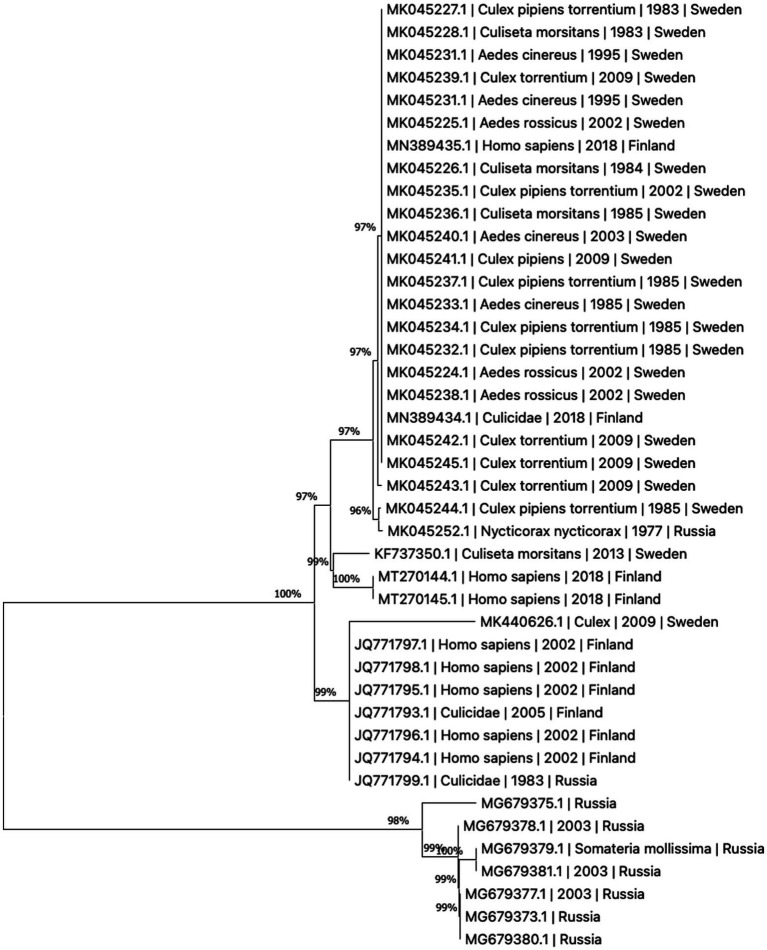
Phylogenetic tree based on whole genome sequences of Sinbis virus (SINV) isolates in Finland, Sweden and Russia. The sequences were downloaded from NCBI Virus (43). Analysis was performed by ClustalW alignment and phylogram constructed by a maximum likelihood algorithm, both implemented in MEGA11 (44).

The present literature is unclear whether the variants of human disease (Ockelbo fever, Pogosta disease, and Karelian fever) represent separate entities caused by different strains or are simply local variants in nomenclature. Though fever and rash are present in all three, some characteristics vary between the diseases, suggesting that they may indeed be separate; such as paresthesia being reported only in Ockelbo disease, while Karelian fever rarely features chronic arthritis or arthralgia (2). Sequence analysis of regional samples has not proven the three diseases to be caused by distinct viral strains, see Figure 2. While human infection tends to be subclinical more often than evident, the ratio between subclinical to clinical infection appears to vary by region, with ratios of 20:1–40:1 reported in Sweden and 17:1 in Finland (27). It is worth noting that in Fennoscandia, SINV is a notifiable disease only in Finland (28). In the clinical setting, diagnosis may be made more difficult by a non-specific array of these symptoms, where the rash might be confused for one caused by rubella virus in Pogosta disease, and parvovirus B19 in Ockelbo disease (2, 30, 45).

Incidence rates also vary between countries, with reported figures ranging from 2.9/100000 for Ockelbo fever, 2.7/100000 for Pogosta disease, and 18/100000 for Karelian fever in their respective regions, see Table 1 (30, 34). Seroprevalence is similar, 2.9% in northern Sweden, and 2.5% for Finland; the studies also found increasing seroprevalence with age (34, 46, 47). The SINV infections are all more commonly observed in late summer to early fall. Pogosta disease features an additional pattern of larger endemic outbreaks every 7 years, a pattern not reported in the other regional variants. This remarkable pattern of cyclical outbreaks held true from the first outbreak in 1974 until 2009, when case numbers, although slightly higher than previous years, were significantly lower than those 597 seven cases reported in 2002 (30, 48). The cyclical pattern has not yet returned, it is not yet known what caused the cyclical pattern, or what caused it to cease. Several previously held theories were disproven by the cycle breaking, however grouse populations, a non-migratory bird and important vertebrate host for SINV in Finland, were at a record low in 2009, and did not recover until 2018, which might offer a partial explanation (32). The pattern may have returned, as a new major outbreak occurred in the fall of 2021, when 556 cases were recorded in Finland (49).

**Table 1:** Epidemiology of mosquito-borne viruses in Fennoscandia summarized.

Virus	First isolate in Fennoscandia^a^	Known distribution	Main symptoms or effects of human disease	Seroprevalence in population (%)	Incidence/year (per 100,000)	Incubation period (days)
SINV	1982 (Sweden)	Norway, Sweden, Finland	Fever, rash, arthralgia	2.5 (Finland, Sweden)	2.7 (Finland)	5–7
INKV	1964 (Finland)	Norway, Sweden, Finland	Fever, influenza-like illness; encephalitis	51 (Finland) 41 (Sweden)	-	-
TAHV	1978 (Norway)	Norway, Sweden	Influenza-like illness; respiratory disease, meningoencephalitis	-	-	3–7
CHATV	2007 (Finland)	Finland	Fever, influenza-like illness; encephalitis	-	-	-
BATV	1985 (Sweden)	Norway, Sweden, Finland	Influenza-like illness	<1 (Norway, Sweden, Finland)	-	-

SINV, Sindbis virus; INKV, Inkoo virus; CHATV, Chatanga virus; BATV, Batai virus; TAHV, Tahyna virus.

a First isolates in mosquitoes.

### Inkoo virus

Inkoo virus (INKV), as well as the later mentioned viruses, all belong to the *Orthobunyavirus* genus, INKV is within the California serogroup, a group of serologically and genetically related orthobunyaviruses, all of which are presumed arboviruses, and many recognized human pathogens (50). INKV seropositivity has been recorded in northern Sweden as being significantly higher in men (46.9%) than in women (34.8%), weighted average 40.9% (51). The equivalent figure in Finland was 51.3% (52). Seroprevalence in Norway has not been extensively studied in humans, though a 1985 survey of seroprevalence in Norwegian soldiers 22% of the recruits tested displayed antibodies to California serogroup viruses (53). High IgG seroprevalence has been reported in reindeer (22). Human disease caused by INKV is often asymptomatic, with evident disease characterized by an influenza-like illness. INKV has been linked to cases of neuroinvasive disease in both adults and children, were the latter appeared to have a more severe form of the disease (23, 54). First isolated in *Aedes communis* and *Ae. punctor* in Finland in 1964, INKV has later been found in *Ae. hexodontus, Culex torrentum, Cx. Pipiens* and *Culiseta morsitans* as well. *Ae. communis* and *Ae. punctor* have been observed to have the capacity to act as vectors (23, 27, 38, 55).

INKV, along with the related Tahyna virus, are two of the most common mosquito-borne California group viruses in Eurasia, and have therefore been recommended for close public health surveillance by the World Health Organization (3) Despite of this, INKV is not highly represented in the literature, with only 27 results in PubMed in the last 20 years.^1^

### Tahyna virus

Tahyna virus (TAHV) is closely related to La Crosse virus (LACV), first isolated in the Ťahyňa and Križany villages in what is now Slovakia in 1958 (56), the first mosquito-borne virus to be isolated in Europe (57, 58), TAHV now occurs in most countries of continental Europe (18, 55), with high TAHV antibody prevalence (60%–80%) in human populations in endemic regions, suggesting it to be widespread (59). Yet, only a few isolates have been produced in Fennoscandia (10, 18, 27, 39). Human disease caused by TAHV, known as ‘Valtice fever’ has been documented to cause an influenza like illness, characterized by fever and respiratory symptoms, and in rare cases central nervous system involvement in the form of meningoencephalitis. The disease appears more common in children than adults (18, 55, 56, 59). An incubation period of 3–7 days has been recorded (60).

According to the literature, cases appear limited to mainland Europe, with no reported cases of human disease caused by TAHV reported within Fennoscandia. Due to limited data, it is unclear whether this variance is due to limited TAHV spread, or factors related to the virus itself. TAHV is mainly spread by *Aedes* spp., principally *Ae. vexans* or *Ae. cantans*, though it has also been isolated in other species, such as *Culiseta annulata* and *Culex modestus*. The anthropophilic nature of *Ae. vexans* has been proposed to account for the high antibody rates found in human populations living in endemic areas (3, 54, 57).

### Chatanga virus

Originally isolated in Russia in 1987, Chatanga virus (CHATV) was first isolated in Fennoscandia from *Aedes* spp. mosquitoes collected in Finland in 2007 (52). It has been conjectured that CHATV may be more widespread than previously indicated, due to its antigenic similarity to the more widely studied INKV, which has been reported to have a significant seroprevalence in endemic regions (52). CHATV is described very little in the literature,^2^ but is suspected to behave similarly to INKV in human disease. Putkuri, et al. reported two patients hospitalized with symptoms of fever, headache and nausea were confirmed to be CHATV infections by plaque reduction neutralization testing (PRNT) (23). The patients showed no signs of neuroinvasive disease, as seen in INKV infection.

### Batai virus

Batai virus (BATV) is notable amongst the European orthobunyaviruses in that, unlike the others, it is not a member of the California serogroup, but rather the Bunyamwera serogroup (61). Originally isolated in Malaysia in 1955, BATV was first isolated in Sweden from *Ae. communis* mosquitoes collected in central Sweden 1983–1985 (38). BATV is one of the most geographically widespread orthobunyaviruses, ranging from Malaysia, and India, to most countries in central Europe. Evidence for Fennoscandic circulation has been reported in Norway, Sweden and Finland, with a seroprevalence of around 1% (61). The role of BATV in human disease is not entirely clear, with some cases of disease similar to other orthobunyaviruses being reported in China, though these were identified to involve naturally occurring reassortments with S and L segments from Bunyamwera virus (2, 62). In Africa and Asia, BATV has been described as a non-specific febrile illness, while European cases have been influenza-like in character (63). Fever, bronchopneumonia, tonsilitis and gastritis have all been associated with human BATV infection in former Czechoslovakia (27, 64).

## Pathophysiology of mosquito-borne viral arthralgia and neuroinvasion

As previously discussed, the morbidity of these viral infections in humans is primarily caused by arthralgia, as well as the potential long-term effects of neuroinvasive disease. In many cases the mechanisms behind these effects are poorly understood and understudied, with research in these areas having been mostly focused on the arthralgia of Chikungunya virus (genus *Alphavirus*) and the neuroinvasive disease of West Nile virus (genus *Flavirus*). (65, 66). Several mechanisms have however been identified. These include direct invasion of the joint, joint involvement by immune complex formation, and immune modulation causing a chronic inflammatory response occurring after the transient viremia of the acute phase of the disease. The specific mechanism varies by virus (66). For alphaviruses, who all share a similar mechanism of infection and replication, much of the inflammation is thought to be caused by pro-inflammatory cytokines and matrix metalloproteinases released by infected macrophages in the articular synovium. An inflammatory cycle is then continued by resident cells, who in are in turn infected themselves (67). In sequence alignment studies, another mechanism has been proposed, by which structural proteins in arthritogenic alphaviruses are able to activate T cells similar to endogenous proteins implicated in rheumatoid arthritis (68).

Neuroinvasive disease requires the virus to evade the innate and adaptive immune response such that it may gain entry to the central nervous system (CNS); this is possible through either the neural route, the olfactory route, or the blood–brain barrier. (66) Neural transmission along the axon has not been associated with any mosquito-borne virus. The olfactory route, through infection of olfactory receptor cells in the nasal cavity, has however been demonstrated for both, alphaviruses such as the Venezuelan equine encephalitis virus and the Semliki Forest virus, and orthobunyaviruses such as the La Crosse virus (69, 70). Invasion of the blood–brain barrier, either by adherence to erythrocytes or by pinocytosis has been observed for several alphaviruses, including the Semliki Forest virus (69). Once within the CNS, neurons serve as the main target cells for both encephalitic alphaviruses and orthobunyaviruses (69). This infection triggers an inflammatory response in the neurons, releasing inflammatory cytokines such as interleukin (IL)-1β, IL-6, and tumor necrosing factor (TNF)-α, potentially exacerbating the neuroinflammation (71).

## Transmission dynamics and vector competence

**Figure 3:**
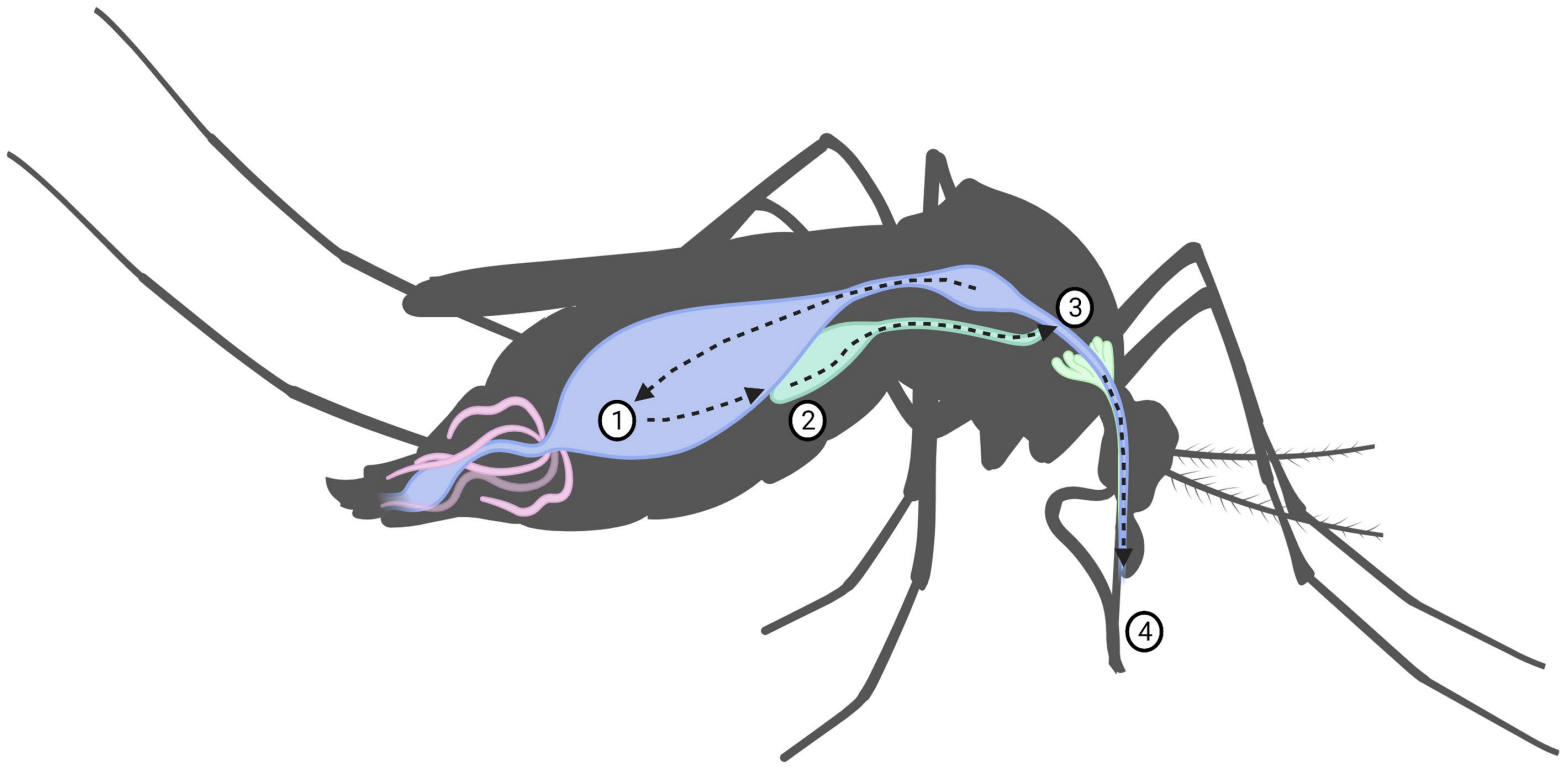
Virus replication in mosquito including the mechanical barriers designated: (1) midgut infection barrier, (2) midgut escape barrier, (3) salivary infection barrier and (4) salivary escape barrier.

For an arbovirus to be successfully transmitted, a complicated series of events and interactions involving the virus, arthropod vector, vertebrate reservoir host and human, all depending on environmental and ecological factors must take place. It is these factors that limit the physiological ability of a species to act as a vector for a specific virus, it is this ability that is known as *vector competence.* In the prospective vector, a series of events of just as complex must take place. Virions acquired by the female mosquito during blood-feeding must pass through the gastro-intestinal system, first entering by passing the midgut infection barrier, through the midgut escape barrier, and into the hemolymph, through which it spreads into the other organs, ultimately into the salivary glands where it may replicate see Figure 3. If it successfully replicates and can pass through the salivary gland escape barrier, it may then be passed onto the vertebrate from which the mosquito takes its next blood meal. If the virus fails to pass through any of these barriers, the mosquito is simply a dead-end vessel for the virus, a so-called *mechanical vector* (58, 72). Because of this, one must keep in mind that a mere virus detection in a species does not necessarily mean that the species is a competent vector. A vector is only truly competent if the ingested virus passes through the organism as previously described such that viremia occurs in, and escapes from, the salivary glands. Thus, vector competence can only truly be determined if viremia in the saliva is measured, detection of virus in any other way might only indicate that the mosquito ingested a blood meal from an infected host (58). Therefore, confirming a species as a vector of a certain virus with certainty is a scientifically rigorous process requiring an infection study of several stages of *in vivo* and *in vitro* study. Vector competence studies have been performed on SINV with wild caught mosquitoes in Sweden (73–75), though no other studies have been performed yet in the rest of Fennoscandia, or on any of the orthobunyaviruses. Another special case is the so-called reservoir host. This occurs when an organism is infected by a vector but shows little susceptibility to the pathological effects of the virus. This organism may then develop sufficient viremia for a new vector to acquire the virus during blood feeding, without developing significant disease itself. Reservoir hosts may then maintain the endemic state of a virus, acting as sources for a virus even if the vectors are not themselves carrying the virus, such as during periods of climate to harsh for the mosquito vectors to survive in aduult form. This creates an ecological system, in which the virus may survive indefinitely (76).

Host-vector transmission as above requires that the infected mosquito was to feed first on an infected host, and then take a second blood-meal from a new host, with enough time in between to develop sufficient viremia itself. There are however a number of viruses capable of being transmitted from the infected female to the next generation (known as *transovarial transmission*) whereby only a single blood-feeding would be sufficient. This will also amplify the effect, as it is not only a single mosquito that is infected, but possibly all of the brood which it lays. This also allows the virus to overwinter in species where the adults do not survive the winter. Of the 18 California serogroup viruses known, 9 have so far been shown to be transovarially transmitted by their mosquito vectors, including the aforementioned La Crosse virus, California encephalitis virus, Jamestown Canyon virus, Tahyna virus, Inkoo virus, as well as the Snowshoe hare virus, Keystone virus, Trivittatus virus, and Morro Bay virus (24, 50). This has also been demonstrated in SINV (77).

### Diagnosis

As previously mentioned, for most of these mosquito-borne viruses, the diagnostic criteria that are to be used when assessing whether to include a suspected infection amongst to differential diagnoses are practically non-existent. These principles of inadequate diagnostic criteria are relevant even outside the scope of Fennoscandian arbovirus infections. The La Crosse virus (LACV) is a major cause of pediatric viral encephalitis in the United States, with a reported case fatality of up to 1.9% of confirmed cases (78). LACV encephalitis has been reported to often be mistaken for herpes simplex (HSV) or meningoencephalitis due to similar case presentations and can so often evade correct diagnosis (23, 78). Other viruses in the same California serogroup, e.g., Jamestown Canyon Virus, have also been reported to cause neuroinvasive disease, though these are reported more rarely, and are studied even less than LACV (23).

Diagnostics should be performed in a two-tiered approach, first testing for common and/or treatable etiologies, as well as those suspected based on specific risk factors. The second tier consists of broader, more invasive tests if previous diagnostic measures remain unsuccessful. Available methods for collecting material for laboratory diagnosis (Polymerase chain reaction; PCR, PRNT, antibody panels etc.) are sampling of blood, CSF by lumbar puncture when involvement of the central nervous system is suspected, serological testing for general malaise, and puncture of vesicles with sampling of exudate in vesicular rashes (79). These tests are then usually sent away for laboratory analysis at larger hospital laboratories. Although simpler kits for viral analysis are available, they are for research use only, and not rated for diagnostic use.

Due to the close genetic and antigenic relationships the orthobunyaviruses form, especially those in the California serogroup, cross reactivity in serology diagnostics makes differentiation between the viruses difficult, necessitating the use of neutralization assays (e.g., PRNT). These assays are not routinely used in clinical practice, except for in certain cases, eg. when certain notifiable diseases are suspected, as they are expensive and usually only available at some university hospitals (22). Some of these infections have well developed serological criteria, such as SINV, in which sensitivities of IgM and IgG enzyme immunoassays were reported as 97.6 and 100%; specificities were 95.2% and 97.6%, respectively (80).

Indeed, in many cases the diagnoses is only possible after the fact, often once an outbreak has already occurred and an increase in similar case presentations appear, a phenomenon observed in the SINV outbreak in Sweden in 2013. IgG may then be used to confirm seroprevalence. (36).

### Treatment

Treatment of the acute infection is of these moboviruses are strictly limited to the symptomatic, as no specific treatment is available. Also, no vaccine or prophylactic medication has of yet been made available (81).

Though most of the human infections caused by these viruses are generally mild in symptoms, the long-term effects may cause significant morbidity. In a follow-up study of patients with serologically confirmed Pogosta disease, only 50% of patients were found to be symptomless 2.5 years after onset (82). In a study conducted in northern Sweden, seropositivity for SINV was found to be an independent predictor of having had a stroke, odds ratio 4.3 (36, 46).

It is not clear why only some patients infected with orthobunyaviruses develop symptoms. In the case of INKV, it has been theorized that underlying disease or trauma may be a prerequisite for causing entry across the blood–brain barrier, whereby neuroinvasive infection may then occur (23).

So far, there has been little research into the treatment of infections caused by these moboviruses. Limited by few cases being identified in the early stages, very little has been done in situations resembling randomized-control trials. As such, not much is known about optimal treatment, as most infections in the literature were treated either symptomatically, or as the more common disease the infection was mistaken for.

## Insights from a clinician’s perspective

When discussing the subject of this review with Fennoscandian colleagues in medicine, not specifically engaged in infectious medicine, the first question is inevitably: “Hold on, are there mosquito-borne diseases around here?”

The true incidence of California serogroup virus infections in Fennoscandia is unknown, largely due to underdiagnosing and lack of surveillance efforts (23). It is therefore impossible to estimate the effects of these viruses in the human population, as many cases are likely to be misdiagnosed, or even not diagnosed at all. One could easily imagine a case of INKV going undetected, as most patients will not seek medical care for a fever without complications. Should a patient in their early 60’s develops arthritis, one could easily imagine that the symptoms would just be thought of as “a part of normal aging” and no more would be thought of that. In comparing the reported seroprevalence with incidence of human disease for SINV, it becomes evident that the disease is either unreported in many areas, or the disease may take a different form itself. With present diagnostic criteria and case reporting, it is not clear which, if any, is closer to the truth.

It is especially important for general practitioners, who are most likely to see these cases first, to pay attention to infections presenting as fever, rash and/or joint symptoms, especially during late summer to early fall, i.e., from August to September in the Northern Hemisphere. As previously discussed, these infections lack clear case criteria, and can often mask as more commonly seen infections, such as parvovirus B19 or other virus infections with rash in younger patients. In these younger patients, mosquito borne infections are generally not considered as readily, due to the reputation many of these infections have of only occurring in women of late middle-age, which is not the case (2, 83). As many of these infections, particularly SINV, generally occur in outbreaks, being aware of how many patients in the area have presented with similar symptoms may be of great value.

## Future perspectives

One must keep an eye open for the possibility of novel moboviruses being established in Fennoscandia, with viruses such as the Usutu virus and antibodies to West Nile virus already having been found in avian hosts in Sweden (84, 85), human cases may appear soon, although no autochthonous cases has been recorded yet. Early detection of invasive moboviruses, would be greatly aided by cooperation between clinicians and other key players, such as medical researchers, entomologists, veterinarians, and policy makers, using a One Health perspective.

Only with increased awareness of the diseases may the morbidity caused by prolonged infections be reduced. These developments, when in place, will also make the healthcare system more adaptable through preparedness, should a previously unknown virus become endemic in the future or indeed, an endemic mobovirus may, e.g., find more potent vectors and/or more suitable climate, and cause outbreaks (12).

## Data availability statement

The original contributions presented in this study are included in the article, further inquiries can be directed to the corresponding author.

## Author contributions

LW, CA, ME, and OL conceived, designed, and coordinated the writing of the whole manuscript. LW and OL contributed to data collection. All authors contributed to critically revised and approved the final version of this manuscript.

## Funding

This work was financially supported by the Formas grant no. 2020-01056, the Basic Science-Oriented Biotechnology Research at the Faculty of Medicine, Umeå University and the Swedish Research Council grant no. 2019-00773.

## Conflict of interest

The authors declare that the research was conducted in the absence of any commercial or financial relationships that could be construed as a potential conflict of interest.

## Publisher’s note

All claims expressed in this article are solely those of the authors and do not necessarily represent those of their affiliated organizations, or those of the publisher, the editors and the reviewers. Any product that may be evaluated in this article, or claim that may be made by its manufacturer, is not guaranteed or endorsed by the publisher.

## Footnotes

^1^PubMed search:" Inkoo OR INKV," results by year: 2002–2022. Retrieved 2022-07-05.

^2^PubMed search "(Chatanga OR CHATV) AND virus," returned 6 results. Retrieved 2022-07-06. Ovid MEDLINE search "(Chatanga OR CHATV) AND virus, returned 2 text results. Retrieved 2022-07-06.
